# Perlecan and the Blood-Brain Barrier: Beneficial Proteolysis?

**DOI:** 10.3389/fphar.2012.00155

**Published:** 2012-08-23

**Authors:** Jill Roberts, Michael P. Kahle, Gregory J. Bix

**Affiliations:** ^1^Sanders-Brown Center on Aging, University of KentuckyLexington, KY, USA; ^2^Department of Molecular and Cellular Medicine, Texas A&M Health Science CenterCollege Station, TX, USA

**Keywords:** perlecan, blood-brain barrier, vascular basement membrane, extracellular matrix, domain V, stroke, brain

## Abstract

The cerebral microvasculature is important for maintaining brain homeostasis. This is achieved via the blood-brain barrier (BBB), composed of endothelial cells with specialized tight junctions, astrocytes, and a basement membrane (BM). Prominent components of the BM extracellular matrix (ECM) include fibronectin, laminin, collagen IV, and perlecan, all of which regulate cellular processes via signal transduction through various cell membrane bound ECM receptors. Expression and proteolysis of these ECM components can be rapidly altered during pathological states of the central nervous system. In particular, proteolysis of perlecan, a heparan sulfate proteoglycan, occurs within hours following ischemia induced by experimental stroke. Proteolysis of ECM components following stroke results in the degradation of the BM and further disruption of the BBB. While it is clear that such proteolysis has negative consequences for the BBB, we propose that it also may lead to generation of ECM protein fragments, including the C-terminal domain V (DV) of perlecan, that potentially have a positive influence on other aspects of CNS health. Indeed, perlecan DV has been shown to be persistently generated after stroke and beneficial as a neuroprotective molecule and promoter of post-stroke brain repair. This mini-review will discuss beneficial roles of perlecan protein fragment generation within the brain during stroke.

## Introduction

A number of neurological diseases such as multiple sclerosis, Alzheimer’s disease, and stroke have been associated with dysfunction of the blood-brain barrier (BBB; Zlokovic, 2008; Yang and Rosenberg, 2011; D’Aversa et al., 2012). The BBB consists of several layers of protection between the blood and brain parenchyma, endothelial cells with specialized junctions and transporters, the vascular basement membrane (BM) composed of extracellular matrix (ECM), and the astrocyte end-feet. While numerous studies have focused on examining the role of tight junctions and transport mechanisms such as ABC transporters (e.g., P-glycoprotein) within the endothelial cells, much less is known about the role of the vascular BM during brain injury and disease.

Prominent ECM components of the vascular BM include fibronectin, laminin, collagen IV, and perlecan, all of which regulate cellular processes and homo- and heterotypic intercellular signaling via interaction with integrins and other ECM receptors (del Zoppo and Milner, 2006; Baeten and Akassoglou, 2011). During development these substances play a critical role in the assembly and stability of BMs, which are located throughout the body and especially important for the proper development of the heart, cartilage, and brain (Yurchenco and Schittny, 1990; Arikawa-Hirasawa et al., 1999; Costell et al., 1999; Giros et al., 2007). During pathological conditions of the central nervous system (CNS), the expression and proteolysis of these ECM components is altered due to an increase in the activity of proteases including matrix metalloproteinases (MMPs), cathepsins, and others (Fukuda et al., 2004). In particular, the heparan sulfate proteoglycan perlecan is proteolytically cleaved within hours following ischemic stroke (Lee et al., 2011). Such proteolysis results in the degradation of the vascular BM and further disruption and dysfunction of the BBB.

While it is clear that such proteolysis has some negative consequences for the BBB, we propose that it also may lead to the generation of ECM protein fragments, including the C-terminal domain V (DV) of perlecan, that potentially have a positive influence on other aspects of CNS health. Indeed, increased production of perlecan DV is observed within the ischemic core and penumbra following experimental stroke (Lee et al., 2011), suggesting it may play a role in the brain’s response to stroke. In this mini-review we will discuss the role of perlecan within the brain and the possible beneficial role of perlecan protein fragment generation following stroke.

## What is the Matrix?

**Figure 1:**
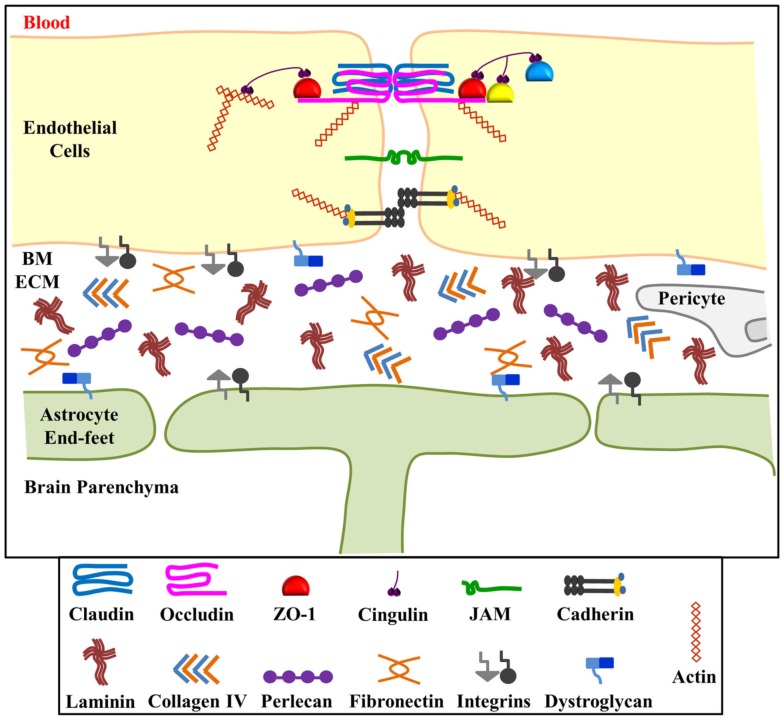
**Schematic representation of the blood-brain barrier (BBB)**. The BBB consists of interendothelial cell tight junctions and adherens junctions, the extracellular matrix components of the basement membrane, pericytes and astrocyte end-feet. The basement membrane extracellular matrix (ECM) components hold endothelial cells and astrocytes in close proximity and contribute to the permeability and stability of the BBB. ECM components include laminin, collagen IV, fibronectin, and perlecan which bind to and signal through integrin (α and β) and dystroglycan (α and β) receptors located on both endothelial cells and astrocytes. Perlecan, and potentially other ECM components of the BBB, are processed into smaller biologically active fragments upon BBB injury. We hypothesize that these fragments, in turn, might have beneficial effects on brain injury recovery. Figure adapted from Huber et al. (2001) and del Zoppo and Milner (2006).

The BBB exists to maintain brain homeostasis and normal neuronal function. The major structures which characterize the BBB are: (1) tight junctions and adherens junctions between adjacent microvessel endothelial cells, which impart a high transendothelial electrical resistance and low paracellular permeability; (2) intact vascular BM; (3) pericytes; (4) astrocyte end-feet (Figure 1). Recent data suggest that the endothelial cell barrier properties are only partly explained by tight junction proteins. Pericytes are important for capillary stabilization and maturation of endothelial cell contacts and astrocyte adhesion is required to regulate the quality of the barrier (Bolton et al., 1998; Wolburg and Lippoldt, 2002; Willis et al., 2004; Dore-Duffy, 2008; Kamouchi et al., 2012). Importantly, stability of the endothelial cell-astrocyte configuration requires an intact vascular BM (Willis et al., 2004).

Basement membranes are sheet-like cell-adherent extracellular matrices that contribute to tissue organization, stability, and differentiation (Yurchenco and Patton, 2009). The vascular BM surrounds vascular endothelia and is composed of the ECM components laminin, collagen type IV, fibronectin, and perlecan (Figure 1). Proper positioning of astrocyte end-feet to the abluminal endothelial surface occurs via the cross-linked network of these ECM components and integrin and dystroglycan cell surface receptors (Willis et al., 2004). While laminin appears to be the primary ECM component required for BM assembly, perlecan plays a critical role in BM maintenance and stability (Arikawa-Hirasawa et al., 1999; Costell et al., 1999). A lack of perlecan *in vivo* has been shown to lead to disrupted BMs and even lethality due to developmental defects (George et al., 1993; Arikawa-Hirasawa et al., 1999; Poschl et al., 2004).

## Perlecan

**Figure 2:**
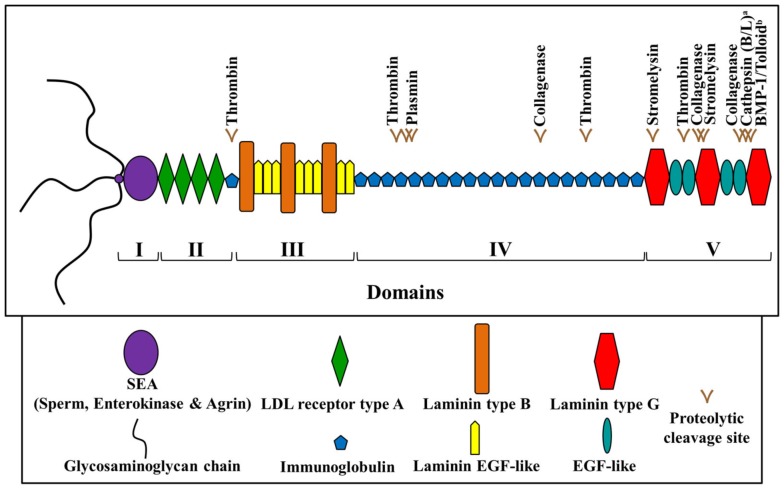
**Schematic diagram of human perlecan**. Perlecan is made up of five domains and contains various predicted and experimentally determined proteolytic (proteases as labeled) cleavage sites. ^a^Saini and Bix (2012); ^b^Gonzalez et al. (2005). Adapted from Farach-Carson and Carson (2007) and Whitelock et al. (2008).

Perlecan (>400 kDa) is a heparan sulfate proteoglycan which contains a multi-domain protein core and three glycosaminoglycan chains at its N-terminus. Its five distinct domains (Figure 2) are known to interact with a wide range of biological molecules, including growth factors and other ECM components, allowing it to mediate cell signaling events controlling migration, proliferation, and differentiation (Whitelock et al., 2008). Perlecan has also long been speculated to serve as an extracellular depot or reservoir for growth factors, potentially released upon ECM disruption (Bix and Iozzo, 2008). Developmental *in vivo* studies have shown that perlecan is of particular importance during cardiovascular, cartilaginous, and neural development. Perlecan-deficient mice demonstrate a complex series of phenotypes which are not confined to one tissue or organ system. Embryos lacking perlecan showed severe chondrodysplasia, life-threatening malformations of the heart outflow tract, as well as impaired telencephalic development (Handler et al., 1997; Costell et al., 1999; Ford-Perriss et al., 2003). Most of the mice survive the very early stages of development, but approximately half die around embryonic day 10.5 (E10.5) because of either malformations of the heart or failure of the nervous system to develop. Those that are born die soon thereafter because of respiratory failure likely due to major skeletal abnormalities present in the ribs.

Studies have shown that perlecan-deficient mice exhibit normal BM formation, but with time BM regions exposed to increased mechanical stress, such as the expanding brain ventricles, deteriorate. This decrease in brain BM integrity leads to neuronal ectopias and exencephaly (Costell et al., 1999). In addition, Giros et al. determined that perlecan influences the size of the ventral and cortical telencephalic structures, as both of these were reduced in perlecan-deficient mice. Giros et al. (2007) speculated that this deficit may be due to insufficient recruitment and/or signaling by the morphogen Sonic hedgehog (SHH) in the basal lamina of the floor plate. High levels of perlecan protein and mRNA expression have also been observed in the developing vascular system, including the cerebral microvasculature (Handler et al., 1997). The process of blood vessel formation is thought to involve activation of growth factors (e.g., FGF and TGFβ), differentiation of smooth muscle cells, inhibition of endothelial cell proliferation, and activation of matrix production (Folkman and Shing, 1992). As mentioned above, perlecan is thought to be an important regulator of growth factor signaling and could modify the behavior of replicative cells by controlling the amount of various growth factors involved in vascular morphogenesis (Handler et al., 1997).

Growth factors which bind to perlecan include vascular endothelial growth factor (VEGF), platelet-derived growth factor (PDGF), transforming growth factor (TGFβ), and in particular the fibroblast growth factor (FGF) family (for review see Whitelock et al., 2008), known regulators of neovascularization. Deguchi et al. (2002) demonstrated that perlecan mediates FGF internalization at the endothelial cells of the BBB and suggest that it may also be an important carrier of FGF secreted from astrocytes. Perlecan, therefore, plays an important role in BBB function via growth factor regulation, as FGF is suggested to be one of the soluble factors necessary for maintaining BBB integrity.

Effects of perlecan on the endothelial cells of the cerebral microvasculature also occur via the integrin and dystroglycan families of matrix adhesion receptors. These receptors regulate cell behavior by transducing extracellular stimuli to intracellular signals, and they form a physical link between the intracellular cytoskeleton and the ECM. They are expressed on endothelial cells and astrocytes and evidence suggests that their expression is perturbed during pathological states of the CNS, including cerebral ischemia. Perlecan (Domain V) is known to bind to α2β1 and α5β1 integrins and to α-dystroglycan (Bix et al., 2004; Ahsan et al., 2011; Wright et al., 2012). Interestingly, when endothelial cells were cultured on single ECM component substrates perlecan not only increased expression of α5β1 integrin, but it did so more than collagen IV and fibronectin (Milner et al., 2008b). This data indicate that perlecan is also able to regulate BBB function by way of receptor expression.

## Perlecan Proteolysis

As mentioned above, perlecan is important for normal neural development and proper functioning of the BBB. What happens to perlecan following brain injury or disease? Is perlecan simply degraded? Perlecan expression has been shown to decrease 43–63% within a few hours following middle cerebral artery occlusion as measured by perlecan immunohistochemistry (Fukuda et al., 2004). Interestingly, however, it is quite likely that this study employed multiple perlecan monoclonal antibodies that collectively could not detect all five perlecan protein domains. For example, the clone A7L6 antibody used in the study is specific for perlecan domain IV. Along this line of reasoning, we propose that perlecan, rather than simply being degraded after brain injury/disease, is being processed into potentially beneficial protein fragments. Such fragments may not have been detected in the Fukuda et al. study due to incomplete immunogenic coverage of the perlecan protein core. However, the Fukuda et al. study importantly determined that following cerebral ischemia perlecan is the most sensitive ECM component (compared to collagen or laminin) to proteolysis in the ischemic core (Fukuda et al., 2004). Indeed, in our own studies, the C-terminal fragment of perlecan, DV, is endogenously and persistently increased following stroke in mice and rats (Lee et al., 2011), sheep, non-human primates, and humans (unpublished data from Bix Lab). Additionally, in rats and mice, the C-terminal fragment of DV, laminin-like globular domain 3 (LG3), is increased (i.e., proteolytically cleaved from full length perlecan) in ischemic tissue following oxygen-glucose deprivation (Saini et al., 2011). DV and LG3 are proteolytically cleaved via cathepsins and BMP-1/Tolloid-like MMPs (Gonzalez et al., 2005), which are also upregulated following stroke.

Using oxygen-glucose deprivation (OGD), an *in vitro* model system reproducing these primary stimuli that injure cells during stroke, our group showed that neurons and pericytes, but not astrocytes, increase their release of LG3. Interestingly however, upon reperfusion all three of these cell types show increased LG3 when stimulated by brief durations of OGD. This is in contrast to prolonged durations of OGD where neurons, pericytes, and brain endothelial cells show decreased LG3 (Saini et al., 2011). Specifically, the source of this increased LG3 following OGD appears to be the result of increased perlecan synthesis and secretion. Additionally, increased cathepsin-L, which appears to be important for LG3 cleavage under normal conditions, and cathepsin-B, which appears to be important for LG3 cleavage following OGD, increase LG3. It is also important to note that the inflammatory mediator interleukin (IL)-1α also increased cathepsin-B and LG3 levels (Saini and Bix, 2012). Collectively, perlecan and its DV and LG3 fragments are increased following stroke, which likely involves multiple cell types.

The findings of increased perlecan and its fragments following stroke led us to hypothesize that these molecules may play a role in stroke outcome. Initially, we demonstrated that strokes in mice that expressed 10% of total normal perlecan levels (perlecan hypomorphs) suffered significantly larger infarcts (and more severe functional deficits) than their wildtype littermates, suggesting that perlecan, and potentially by extension, DV/LG3, play a role in the brain’s response to transient ischemia. We then demonstrated robust benefits of administering exogenous human recombinant DV following experimental stroke in rodents. Importantly, most of these benefits were replicated in two different species – rats and mice, using two different stroke models – a tandem ipsilateral common carotid and middle cerebral arteries occlusion (CCA/MCAo) model and the endothelin (ET)-1 injection model. Benefits of DV administration following stroke include: (1) neuroprotection, (2) angiogenesis, (3) rescue of stroke-affected motor function, and (4) modulation of astrocyte activity and reduction of glial scar.

(1) *Neuroprotection*: When administered by intraperitoneal (i.p.) injection beginning 24 h after injury and then every-other day following, DV decreased ischemic lesion size (as measured by TTC and H&E staining) and reduced immunoreactivity of TUNEL and Caspase-3 cleavage (markers of apoptosis; Lee et al., 2011). Furthermore, LG3 was neuroprotective *in vitro* to fetal cortical neurons exposed to OGD, when compared to PBS treated controls, as evident by a 300% viability increase and decreased immunohistochemical staining of Caspase-3 (Saini et al., 2011). Interestingly, DV and LG3 were also neuroprotective to Amyloid-β mediated toxicity (believed to play a role in Alzheimer’s disease), via α_2_- and α_v_-β_1_ integrins, in human cortical neurons (Wright et al., 2012). Collectively, these findings indicate that perlecan DV/LG3 is neuroprotective following stroke, OGD, and Amyloid-β stressors, suggesting that perlecan and its fragments may also be neuroprotective to other CNS diseases and insults as well.
(2) *Angiogenesis*: Post-stroke exogenously administered DV induced the secretion of VEGF from brain endothelial cells, as evidenced by ELISA and Western blot. This release in VEGF occurred following DV association with its endothelial cell α5β1 receptor resulting in the formation of new blood vessels, as evidenced by immunohistochemistry. Although α5β1 integrin is largely absent in the brain’s microvasculature after development, it is re-expressed following stroke and is increased in brain endothelial cells in direct response to perlecan, thereby facilitating an angiogenic cascade (Milner et al., 2008a). Mechanistically, DV appears to activate PI3K-Akt, MEK-ERK, eIF4E, c-Jun, and HIF-1α downstream from its α5β1 integrin receptor to induce VEGF synthesis and release from brain endothelial cells (Clarke et al., submitted manuscript). Importantly, this DV-induced increase in VEGF and angiogenesis does not cause further disruption of the BBB (Lee et al., 2011). The mechanisms responsible for this apparent paradox – VEGF not causing further disruption of the BBB – have not yet been fully elucidated but likely involve indirect action by another cell type, potentially astrocytes.
(3) *Rescue of stroke-affected motor function*: In both wildtype and perlecan hypomorph animals, DV restored (to pre-stroke levels) stroke-affected motor function as measured by the vibrissae-elicited paw placement test and the cylinder test. This functional rescue was stable 2 weeks after injury (Lee et al., 2011).
(4) *Modulation of astrocyte activity and reduction of glial scar*: Exogenous DV administration decreases astrogliosis *in vitro*, and the post-stroke secretion of glial scar proteins neurocan and phosphacan *in vivo* (Al-Ahmad et al., 2011). This reduction of glial scar is a significant finding as glial scar can be a physical and chemical barrier to neurorepair following stroke. We have also shown that DV has direct effects on astrocytes and influences their activity *in vitro* (Al-Ahmad et al., 2011). For example, DV inhibits the proliferation and promotes the adhesion, migration, stellation morphology, and secretion of NGF from astrocytes.

Importantly, DV-induced neuroprotection and rescue of motor function were also observed following a permanent photothrombosis model of stroke conducted in a laboratory distinct from our own (Clarkson et al., submitted manuscript). In this model, DV administration was effective when treatment was initiated 6 h, but not 8 or 12 h, following injury. This earlier therapeutic window may be accounted for by the faster evolution of infarct size in the photothrombotic model compared to CCA/MCAo and ET-1 models.

In summary, we suggest that perlecan, and potentially other ECM components of the BBB, is not simply degraded after stroke, but is actively processed, resulting in the endogenous generation of DV and LG3. DV and LG3, in turn, appear to be important to the brain’s response to stroke inasmuch as their deficiency results in worsened stroke outcomes. Furthermore, when DV is administered after stroke, it provides various benefits including neuroprotection, angiogenesis, rescue of stroke-affected motor function, and reduction of astrogliosis and glial scar. Therefore, in conditions involving BBB dysfunction, we suggest that more attention be given to the bioactive fragments of the ECM that are generated, as the ECM is not simply degraded into inactive molecules. Ultimately, further investigation may identify other biologically significant BBB ECM fragments, expanding a relatively unexplored venue of BBB research, and potentially leading to novel CNS therapies.

## Conflict of Interest Statement

The authors declare that the research was conducted in the absence of any commercial or financial relationships that could be construed as a potential conflict of interest.
